# Risk for Severe Illness and Death among Pediatric Patients with Down Syndrome Hospitalized for COVID-19, Brazil

**DOI:** 10.3201/eid2901.220530

**Published:** 2023-01

**Authors:** Char Leung, Li Su, Ana Cristina Simões-e-Silva, Luisamanda Selle Arocha, Karina Mary de Paiva, Patricia Haas

**Affiliations:** Author affiliations: University of Leicester, Leicester, England, UK (C. Leung); University of Cambridge, Cambridge, England, UK (C. Leung, L. Su, A.C. Simões-e-Silva, L.S. Arocha); Universidade Federal de Minas Gerais, Belo Horizonte, Brazil (A.C. Simões-e-Silva); University of Cambridge, Cambridge (L. Selle Arocha); Universidade Federal de Santa Catarina, Florianopolis, Brazil (K.M. de Paiva, P. Haas)

**Keywords:** COVID-19, respiratory infections, severe acute respiratory syndrome coronavirus 2, SARS-CoV-2, SARS, coronavirus disease, zoonoses, viruses, coronavirus, Down syndrome, Brazil

## Abstract

Down syndrome is the most common human chromosomal disorder. Whether Down syndrome is a risk factor for severe COVID-19 outcomes in pediatric patients remains unclear, especially in low-to-middle income countries. We gathered data on patients <18 years of age with SARS-CoV-2 infection from a national registry in Brazil to assess the risk for severe outcomes among patients with Down syndrome. We included data from 14,684 hospitalized patients, 261 of whom had Down syndrome. After adjustments for sociodemographic and medical factors, patients with Down syndrome had 1.8 times higher odds of dying from COVID-19 (odds ratio 1.82, 95% CI 1.22–2.68) and 27% longer recovery times (hazard ratio 0.73, 95% CI 0.61–0.86) than patients without Down syndrome. We found Down syndrome was associated with increased risk for severe illness and death among COVID-19 patients. Guidelines for managing COVID-19 among pediatric patients with Down syndrome could improve outcomes for this population.

Described by John Langdon Down in the 19th Century, Down syndrome is a birth defect caused by a random error in cell division during meiosis that results in an additional full or partial copy of chromosome 21. Down syndrome is rare but is the most common chromosomal disorder; the global estimated birth prevalence is 14 cases/10,000 live births, and prevalence in Brazil is 4 cases/10,000 live births (*1*). The lower prevalence in Brazil might be because of the difference in the maternal age profile (*1*). Nevertheless, death among persons with Down syndrome in Brazil has increased in recent years, particularly among children (*2*). Socioeconomic and regional differences in the quality of and access to healthcare, particularly in the North and Northeast regions, might explain increased death rates (*2*,*3*). Furthermore, the government of Brazil has proposed healthcare guidelines for persons with Down syndrome, but compliance remains poor (*4*).

Down syndrome is characterized by anatomic abnormalities and intellectual disabilities. Persons with Down syndrome are more prone to chronic diseases, including visual impairment (prevalence 73%), thyroid disease (50%), congenital heart disease (25%), hypoacusis (27%), obesity (22%), osteoporosis (20%), and epilepsy (8%) (*5*). Because of immune disturbances, Down syndrome patients are more susceptible to respiratory tract infections and acute respiratory distress syndrome (ARDS) (*6*). Congenital heart defects and respiratory infections remain the most common causes of death and hospitalization among persons with Down syndrome (*7*,*8*). In children with Down syndrome, <80% of all hospitalizations and intensive care unit (ICU) admissions result from lower respiratory tract infections, and <29% of deaths are associated with pneumonia, influenza, and aspiration (*9*).

Patients with Down syndrome might be at higher risk for COVID-19–related death because they are more susceptible to respiratory failure, a major cause of death among COVID-19 patients. In addition, common underlying conditions among persons with Down syndrome, such as cardiovascular disease and obesity, have been identified as independent risk factors for COVID-19–related death (*10*). Despite these factors, studies of SARS-CoV-2 infection in children and adolescents with Down syndrome remain rare, and findings are limited. One case–control study found low mortality rates in pediatric patients with COVID-19 overall in high and low-to-middle income countries, despite more severe COVID-19 clinical manifestations among patients with Down syndrome (*11*). The same study reported higher mortality rates for adults with Down syndrome than those without, but because of limitations of the data, the study did not find an association between pediatric Down syndrome and risks for COVID-19–related death (*11*). 

To assess whether pediatric patients with Down syndrome are at higher risk for severe COVID-19 outcomes, we conducted a cohort study by using propensity score matching to reduce confounding from underlying conditions associated with Down syndrome. We used a nationwide database in Brazil to examine whether pediatric Down syndrome was associated with increased risk for severe COVID-19 outcomes among hospitalized patients.

## Methods

### Study Cohort

The study population included persons registered in the Severe Acute Respiratory Syndrome Database of Sistema de Informacao de Vigilancia Epidemiologica da Gripe (SIVEP-Gripe; https://painel-sivep-gripe.herokuapp.com), a nationwide database managed by the government of Brazil (*2*). SIVEP-Gripe was developed for severe acute respiratory syndrome surveillance related to influenza and other respiratory viruses during the influenza A(H1N1) pandemic in 2009. Patients who have a reportable disease and are admitted to public or private hospitals are registered in SIVEP-Gripe. When the 2020 pandemic began, COVID-19 was declared a reportable disease and incorporated into the surveillance network. SIVEP-Gripe is the primary source of information on COVID-19 hospitalizations and deaths in Brazil. Basic demographic and medical data were systematically registered in a predetermined form used for severe respiratory disease hospitalizations; data were verified by the medical practitioner at the point of care.

On November 26, 2021, we collected data on all COVID-19 cases registered in SIVEP-Gripe during March 5, 2020–November 22, 2021. We included cases in our study that met all these criteria: PCR positive for SARS-CoV-2; patient recovered or died; and patient was <18 years of age. We excluded data for patients who died of causes other than SARS-CoV-2 infection. We categorized patients as Down syndrome or non–Down syndrome, according to the SIVEP-Gripe database, where Down syndrome is reported by clinical providers on a standardized registry form.

### Data Sources and Measurement

We collected covariate data from SIVEP-Gripe, including sociodemographic factors and clinical characteristics. These data were age, sex, clinical endpoint (discharge or death), time to recover (i.e., time from admission to discharge), ethnicity (Caucasian, Asian, Hispanic/African, or Indigenous), location by region, need for ventilation, ICU admission, vaccination (against influenza and SARS-CoV-2), use of antiviral drugs, and signs and symptoms (including asymptomatic). Data also included underlying conditions, such as cardiovascular, hematologic, liver, renal, pulmonary, and neurologic diseases; asthma; diabetes; immunocompromise; and obesity. 

Patients self-identified sex and ethnicity. We included ethnicity to reflect racial disparities in healthcare access. Signs, symptoms, and underlying conditions referred to those noted at symptom onset or admission. Except for low oxygen saturation (<95%), other signs and symptoms were assessed by certified medical practitioners. We included these signs and symptoms because they are predictors of in-hospital COVID-19 death (*12*). Location referred to North, Northeast, Southeast, Center West, and South regions of Brazil, which we included to reduce bias due to geographic disparities in healthcare access. Other clinical covariates were recorded during the clinical course. Antiviral drugs referred to those used against influenza, such as oseltamivir and zanamivir. Respiratory viral infections were confirmed by PCR tests for influenza, respiratory syncytial virus, human parainfluenza virus, adenovirus, metapneumovirus, bocavirus, rhinovirus, enterovirus, and other coronaviruses that do not generally cause ARDS (namely NL63, OC43, 229E, and HKU1), but that should not be ignored in high-risk populations (*13*); thus, we included them in the analysis.

Not all variables had complete data. For missing data on signs, symptoms, or underlying conditions, we assumed the clinical condition to be absent, following the approach used in a previous study that analyzed the same database (*14*). To reduce age-related selection bias, we calculated age as the difference between the date of birth and the date of symptom onset, rather than the self-reported age. We considered cases without date of birth as missing data and excluded them.

### Outcome and Comparison Group Definitions

The primary outcome was whether Down syndrome is associated with increased risk for in-hospital death, measured by odds ratio (OR) for mortality. The secondary outcome was whether Down syndrome is associated with increased risk for severe illness, measured by hazard ratio (HR) for recovery. We adjusted both outcomes for demographic factors, underlying conditions other than Down syndrome, and intervention.

### Statistical Analysis

To compare descriptive statistics between Down syndrome and non–Down syndrome cohorts, we used Mann-Whitney or Student *t*-tests for continuous variables, such as age and time to recovery, depending on the normality condition. We used Fisher exact rather than χ^2^ tests for dichotomous variables because χ^2^ is an approximate test. For the primary outcome, we calculated the OR for death by using a multivariable logistic regression model. For the secondary outcome, we calculated HR for time to recovery by using a multivariable Cox regression model. We assessed the assumption of proportional hazard by using Grambsch-Therneau test and modified the regression model to meet this assumption if the test result indicated any violations.

For both primary and secondary outcomes, we adopted the forward variable selection procedure for all regression models and used p<0.1 as the threshold and the expected (E) value for sensitivity analysis (*15*). In brief, E value is the minimum strength of association that an unmeasured confounder would need to have with both the case and the control group to fully explain away a specific exposure–outcome association, conditional on the covariates (*15*). Because signs, symptoms, ICU admission, and ventilation are mediators rather than confounders, we removed them from the regression analysis. We chose the Southeast region as the reference for the location variables because healthcare is generally more accessible in this region. We chose Hispanic/African as the reference for ethnicity because those ethnicities represent the largest ethnic group in the country. For the logistic regression model, we used the area under the receiver operating characteristic curve (AUC) to assess the goodness-of-fit. For the Cox regression model, we used the concordance index to assess the goodness-of-fit. We performed all calculations in R version 4.1.1 (The R Foundation for Statistical Computing, https://www.r-project.org) by using MatchIt and survival packages. We considered p<0.05 statistically significant.

Because some variables had missing data, we created an additional category for missing data in categorical variables that allowed for nonrandom missingness. For quantitative variables, we excluded cases with missing data from the corresponding statistical analysis because no reliable information was available for imputation. Neither Brazil nor the United Kingdom required ethics approval for this study because we used de-identified, publicly available data. 

## Results

**Figure 1:**
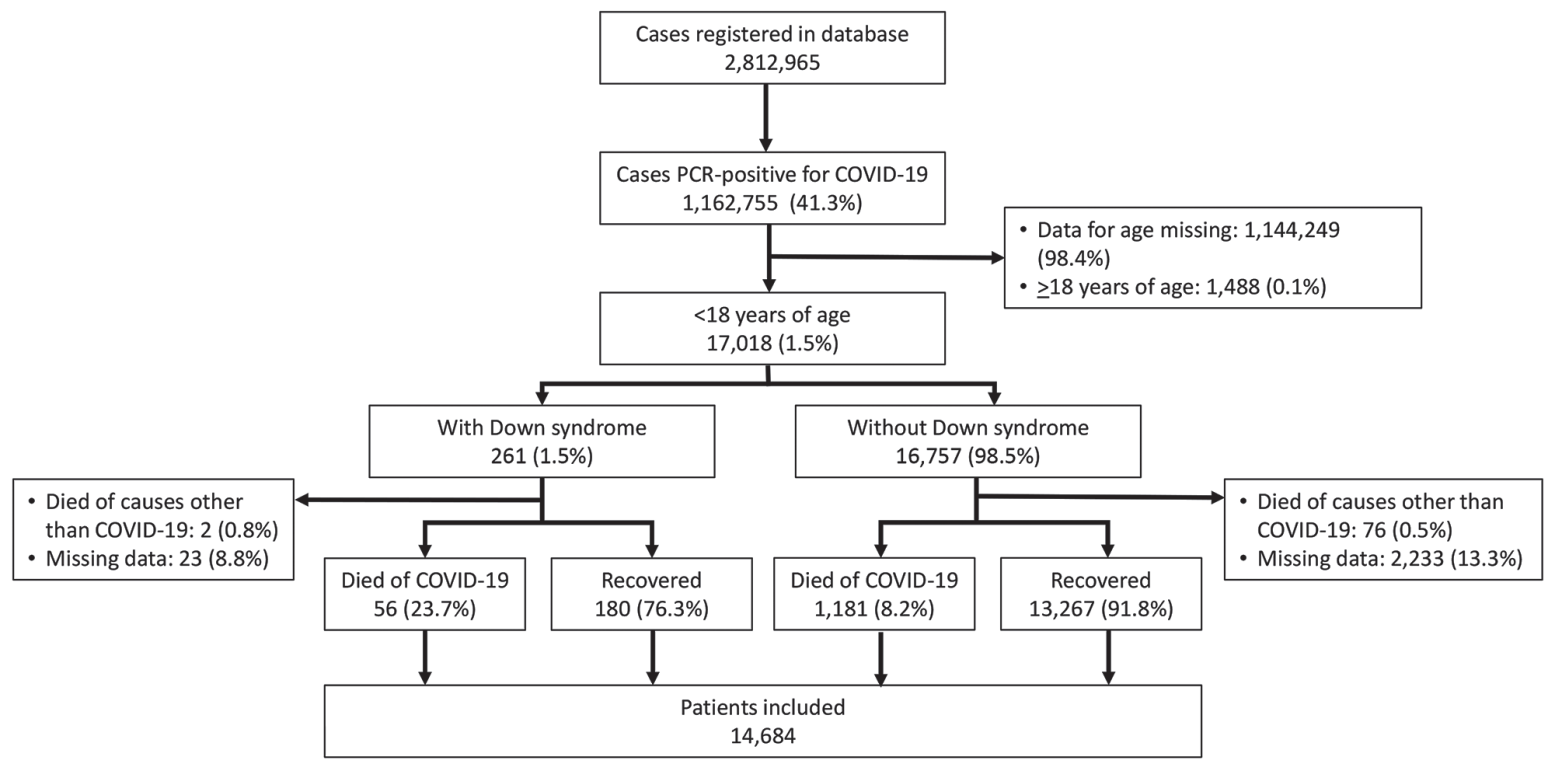
Flowchart of case inclusion in a study of risk for severe illness and death among pediatric patients with Down syndrome hospitalized for COVID-19, Brazil. We used publicly available data from COVID-19 cases registered in the Severe Acute Respiratory Syndrome Database of Sistema de Informacao de Vigilancia Epidemiologica da Gripe (SIVEP-Gripe; https://painel-sivep-gripe.herokuapp.com), a nationwide database managed by the government of Brazil.

A total of 2,812,965 cases were registered in the SIVEP-Gripe, of which 1,162,755 (41.3%) were PCR positive for SARS-CoV-2 (Figure 1). Among those patients, 17,018 (1.5%) were <18 years of age, 1,144,249 (98.4%) were >18 years of age, and 1,488 (0.1%) had missing age data. Among 17,018 patients <18 years of age, 261 (1.5%) had Down syndrome and 16,757 (98.5%) did not, 78 (0.5%) died of causes other than SARS-CoV-2 infection, and 2,256 (13.3%) had missing outcomes. Consequently, the study included a total of 14,684 (86.3%) cases that met all selection criteria and had COVID-19 diagnosed by PCR during March 5, 2020–November 22, 2021.

Among 14,684 patients in the study, 236 (1.6%) had Down syndrome and 14,448 (98.4%) did not (Table). The sample was well balanced for sex (p = 0.237) and median age (p = 0.670) between the 2 groups. We noted no significant difference in ethnicity among the cohort, for Asian (p>0.999) or Indigenous (p>0.999) persons, and for those missing data (p = 0.238). The in-hospital case-fatality rate for patients with Down syndrome was 23.7% and was 8.2% for patients without Down syndrome, and the difference was highly significant (p<0.001). The Down syndrome group also had a longer median time to recover (8.5 days vs. 5 days; p<0.001). Patients with Down syndrome had more signs and symptoms of severe COVID-19 clinical course than patients without Down syndrome, including dyspnea (59.8% vs. 48.9%; p = 0.001), low oxygen saturation (58.9% vs. 37.4%; p<0.001), and respiratory discomfort (59.8% vs. 45.8%; p<0.001). Not surprisingly, patients with Down syndrome were more prone to health conditions, most notably cardiovascular disease (35.6% vs. 3.5%; p<0.001) and immune disorders (6.78% vs. 3.55%; p = 0.013). Furthermore, patients with Down syndrome required more advanced healthcare, evidenced by the higher rates of ICU admission (47.5% vs. 27.0%; p<0.001) and mechanical ventilation (67.4% vs. 44.5%; p<0.001).

**Table:** Characteristics of patients with and without Down syndrome in a study of risk for severe illness and death among pediatric patients hospitalized for COVID-19, Brazil*

Characteristics	No. participants		p value
	With Down syndrome, n = 236	Without Down syndrome, n = 14,448	
Median age, y (IQR)	3.4 (0.6–12.4)	3.6 (0.7–11.5)	0.670
Sex, %	N = 236	N = 14,440	
M	50.0	53.9	0.237
F	50.0	46.1	
Died, no. (%)	56 (23.7)	1,181 (8.2)	<0.001
Median time to recover, d (IQR)	8.5 (4.0–18.0), n =172	5.0 (3.0–10.0), n = 11,919	<0.001
Region, no. (%)			
North	17 (7.2)	907 (6.3)	0.501
Northeast	42 (17.8)	3,350 (23.2)	0.052
Southeast	100 (42.4)	6,908 (47.8)	0.101
Center West	23 (9.7)	1,330 (9.2)	0.734
South	54 (22.9)	1,953 (13.5)	<0.001
Ethnicity, no. (%)	n = 191	n = 11,210	
Caucasian	107 (56.0)	4,894 (43.7)	0.001
Asian	1 (0.5)	87 (0.8)	>0.999
Hispanic	82 (42.9)	6,150 (54.9)	0.001
Indigenous	1 (0.5)	79 (0.7)	>0.999
Missing	42 (19.1), n = 236	3,238 (22.4), n = 14,448	0.238
Signs and symptoms, no. (%)			
Asymptomatic	1 (0.4)	60 (0.4)	>0.999
Abdominal pain	13 (5.5)	883 (6.1)	0.891
Anosmia	2 (0.8)	304 (2.1)	0.248
Ageusia	1 (0.4)	297 (2.1)	0.097
Coryza	18 (8.1)	1,470 (10.2)	0.328
Cough	134 (56.8)	8,909 (61.7)	0.138
Diarrhea	48 (20.3)	1,979 (13.7)	0.006
Dyspnea	141 (59.7)	7,059 (48.9)	0.001
Fatigue	23 (9.7)	1,250 (8.7)	0.559
Fever	163 (69.1)	9,676 (67.0)	0.530
Headache	6 (2.5)	800 (5.5)	0.043
Myalgia	1 (0.4)	387 (2.7)	0.023
Oxygen saturation <95%	139 (58.9)	5,400 (37.4)	<0.001
Respiratory discomfort	141 (59.7)	6,612 (45.8)	<0.001
Sore throat	31 (13.1)	1,941 (13.4)	>0.999
Vomiting	41 (17.4)	2,447 (16.9)	0.861
Other symptoms	77 (32.6)	5,189 (35.9)	0.306
Underlying conditions, no. (%)			
Cardiovascular disease	84 (35.6)	505 (3.5)	<0.001
Hematologic disease	4 (1.7)	274 (1.9)	>0.999
Liver disease	2 (0.8)	68 (0.5)	0.311
Asthma	13 (5.5)	1,050 (7.3)	0.374
Diabetes	2 (0.8)	286 (2.0)	0.337
Neurologic disease	20 (8.5)	805 (5.6)	0.063
Pulmonary disease	11 (4.7)	301 (2.1)	0.018
Immunocompromised	16 (6.8)	513 (3.6)	0.013
Renal disease	6 (2.5)	189 (1.3)	0.137
Obesity	6 (2.5)	324 (2.2)	0.658
Intervention, no. (%)			
Antiviral against influenza	30 (12.7)	1,559 (10.8)	0.341
ICU admission	112 (47.5)	3,904 (27.0)	<0.001
Ventilation	159 (67.4)	6,426 44.5)	<0.001
Influenza vaccinate	18 (7.6)	981 (6.8)	0.601
COVID-19 vaccine	3 (1.3)	81 (0.6)	0.153

*ICU, intensive care unit.

**Figure 2:**
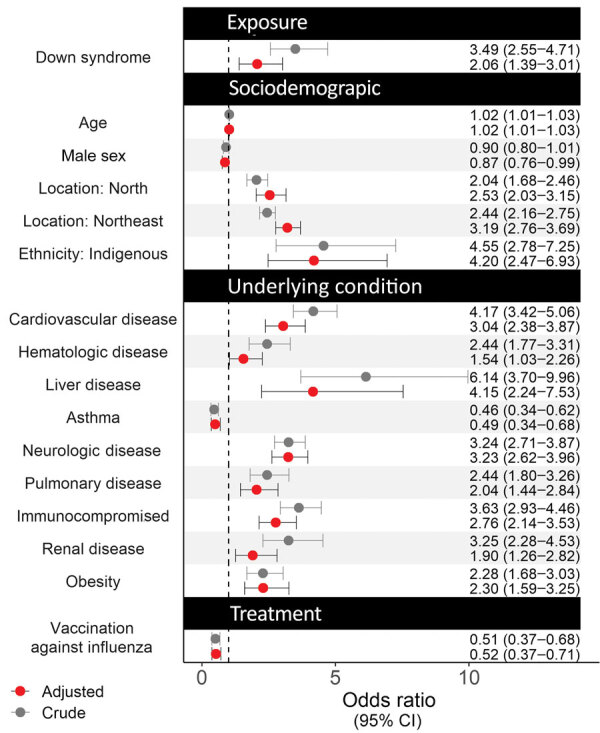
Crude and adjusted odds ratios for various factors associated with risk for severe illness and death among pediatric patients with Down syndrome hospitalized for COVID-19, Brazil. We calculated odds ratios by using logistic regression. Circles indicate odds ratios; error bars indicate 95% CI; dotted vertical line indicates the null hypothesis of odds ratio being equal to 1.

After the adjusting for demographic and clinical factors, multivariable logistic regression suggested that patients with Down syndrome had higher risk for in-hospital death (adjusted OR [aOR] 2.06, 95% CI 1.39–3.01) (Figure 2). Adjusted factors were cardiovascular diseases (aOR 3.04, 95% CI 2.38–3.87), neurologic diseases (aOR 3.23, 95% CI 2.62–3.96), renal diseases (aOR 1.90, 95% CI 1.29–3.01), liver diseases (aOR 4.15, 95% CI 2.24–7.53), and obesity (aOR 2.30, 95% CI 1.59–3.25).

The difference in the crude and adjusted OR for location and ethnicity variables might not reflect confounding effects because we calculated aOR by using a reference group, giving different interpretations. For example, the crude OR for North means that patients from that region had 2 times the odds of death compared with persons from other regions, whereas the aOR showed that patients in the North had ≈2.7 times the odds for death compared with persons in the Southeast region, the reference group.

The AUC was 0.75 (95% CI 0.73–0.75), indicative of accuracy. ORs related to COVID-19 death were usually ≈2 (*16*), close to the computed E values (Appendix Table 1). This result indicates fair robustness of our results for the influence of unmeasured confounders.

**Figure 3:**
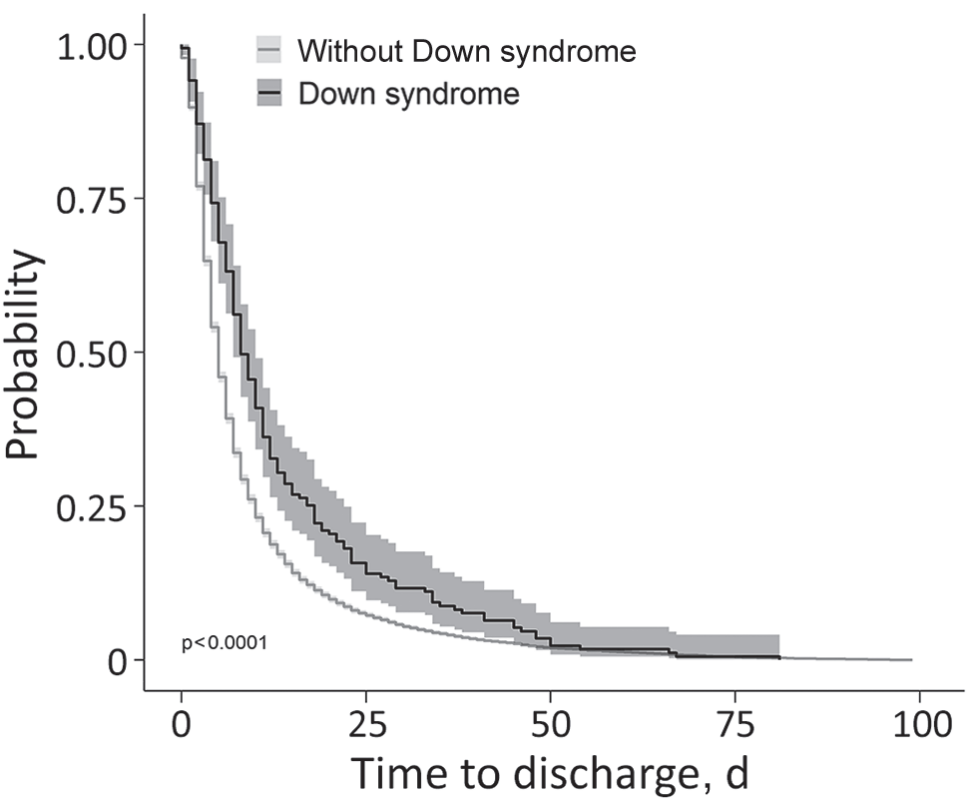
Kaplan-Meier curves for probability of recovery in a study of risk for severe illness and death among pediatric patients with Down syndrome hospitalized for COVID-19, Brazil. The log-rank test suggested a difference in the 2 survival curves (p<0.001), indicating that patients with Down syndrome had a lower probability of recovery during the first month of hospitalization than patients without Down syndrome. Gray shading around lines represents 95% CI.

**Figure 4:**
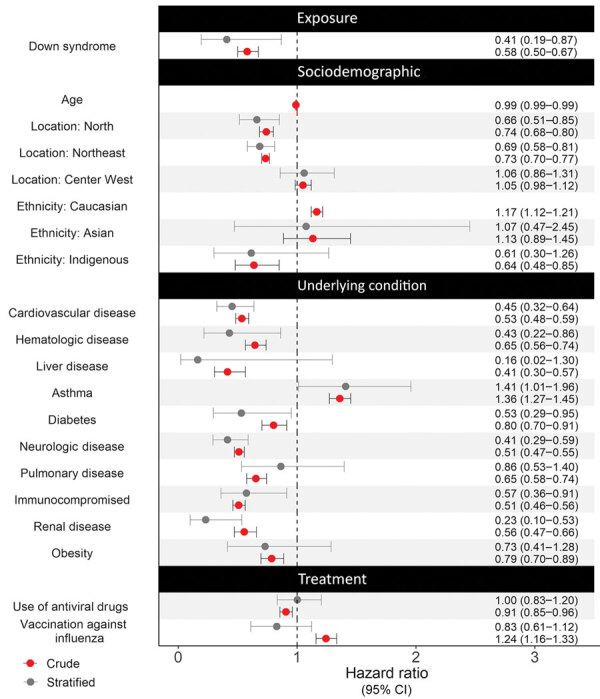
Crude and stratified hazard ratios for various factors associated with risk for severe illness and death among pediatric patients with Down syndrome hospitalized for COVID-19, Brazil. We calculated hazard ratios by using Cox regression. Circles indicate hazard ratio; error bars indicate 95% CI; dotted vertical line indicates the null hypothesis of hazard ratio being equal to 1.

For time to recovery, we created Kaplan-Meier curves for patients with and without Down syndrome (Figure 3). The log-rank test suggested a difference in the 2 survival curves (p<0.001), indicating that patients with Down syndrome had a lower probability of recovery during the first month of hospitalization than patients without Down syndrome. Grambsch-Therneau test results showed that proportional hazard assumption of the multivariable Cox regression model was violated (p<0.001). We stratified the variables for age and Caucasian, based on p values from the Grambsch-Therneau test, and found p = 0.140 in the stratified model (Figure 4). After adjusting for demographic and clinical factors, patients with Down syndrome had 59% longer time to recovery (adjusted HR [aHR] 0.41, 95% CI 0.19–0.97). We also noted statistically significant associations between other underlying conditions and longer time to recovery. Patients with renal disease had 77% longer time to recovery (aHR 0.23, 95% CI 0.10–0.53), those with neurologic disease had 59% (adjusted HR 0.41, 95% CI 0.29–0.59) longer, those with hematologic disease had 57% (aHR 0.43, 95% CI 0.22–0.86) longer, and those with cardiovascular disease had 55% (aHR 0.45, 95% CI 0.32–0.64) longer.

The concordance index was 0.59 (95% CI 0.57–0.61), indicating that the Cox regression model was adequate. Adjusted HR of risk factors related to time to recovery in COVID-19 patients was usually 1.2–1.7 (*17*), smaller than most of the computed E values (Appendix Table 2), indicative of fair robustness of our results.

## Discussion

After adjusting for demographic and medical factors, we found pediatric patients with Down syndrome hospitalized for COVID-19 had higher risk for severe illness and death than those without Down syndrome. We observed higher mortality rates (23.7%) among Down syndrome patients than in a previous case–control study (*11*). In that study, the authors reported a 6.7% mortality rate among 328 children with Down syndrome from low-to-middle income countries (*11*), but their result might be prone to selection bias because all controls were from the United States. Mortality rates reported in that study might also be lower than we observed because of socioeconomic inequality and vulnerability in Brazil. Despite efforts to ensure access to health services for persons with disabilities (*18*), inequality in healthcare access continues in Brazil. We noted that pediatric Down syndrome patients hospitalized with COVID-19 in regions of Brazil with a low socioeconomic profile, such as the North and Northeast, had ≈3–4 times the odds for death and ≈30% longer time to recovery than those in the South region (Figures 2, 4). Furthermore, inequality in access to heart surgery to treat Down syndrome–related heart defects also might explain the higher in-hospital case-fatality rate observed in our study because cardiovascular disease is an independent risk factor for COVID-19–related in-hospital death. Even in southern Brazil, where healthcare is more accessible, recent literature indicates only one quarter of patients with Down syndrome have undergone heart surgery (*19*). Nevertheless, we found that Down syndrome was a risk factor for severe COVID-19 after we adjusted for cardiovascular disease; having undergone heart surgery implies the presence of cardiovascular disease.

Beyond Brazil, several factors specific to Latin America might explain why Down syndrome could be a risk factor for severe COVID-19 outcomes. First, quality healthcare might be out of reach for families of children with Down syndrome because they often are ostracized by the community due to religiously motivated social perceptions of Down syndrome. For instance, one study reported medical practitioners in Ecuador rarely diagnose Down syndrome in an empathetic manner (*20*), which can lead parents to distrust the healthcare system and discourage them from seeking professional help. Second, some families lack the financial ability to access healthcare for COVID-19 even when they live in a geographic area where healthcare is accessible. Therefore, the increased risk for death related to the lack of healthcare might not reflect geographic variables in our study but instead might be associated with Down syndrome status. This problem does not only exist in Brazil; a sizeable portion of the population in Latin America has no access to any kind of health insurance (*21*). Third, Latin America remains one of the least vaccinated areas in the world; barely 30% of the population has been vaccinated against COVID-19 (*22*). Although Latin America has only 8.4% of the world’s population, the region contributed 20% of confirmed global COVID-19 cases and 30% of deaths (*22*). Vaccination rate was low in the pediatric population in our study, and no information was available regarding differences in vaccination rates among children with and without Down syndrome. Finally, ICU admission and ventilation might be less available to children with Down syndrome in low-income countries such as Brazil.

Increased risk for severe illness and death among patients with COVID-19 and Down syndrome intensifies the burden of Down syndrome, which already has greater effects on society in Latin America because resources are scarce. Prenatal diagnostic testing is not affordable for many in Latin America; noninvasive prenatal testing costs 238% and amniocentesis costs 68% of the average monthly income in Brazil (*23*). As of 2014, Paraguay had only 1 laboratory for genetic testing, but many samples were sent to Chile, Brazil, or Argentina for testing (*24*), further straining the resources in these countries.

No literature confirming a biological link between Down syndrome and COVID-19 outcomes is available. Because cytokine release syndrome is a leading cause of COVID-19 deaths, we speculate that increased interleukin-6 (IL-6) production in children with Down syndrome (*25*) could increase risk for death and that elevated IL-6 results from altered immune response to viral infection in patients with Down syndrome, as noted with influenza (*26*). We also hypothesize that patients with Down syndrome might be more susceptible to poor COVID-19 outcomes because the *TMPRSS2* gene, a serine protease for SARS-CoV-2 spike protein priming for viral host cell entry, is located on the *21q22.3* gene (*27*), a critical part of the Down syndrome region.

Several studies have reported on SARS-CoV-2 infection among Down syndrome patients, but those studies focused on the general population or adults. Nonetheless, those studies generally noted more severe COVID-19 in persons with Down syndrome, aligning with our work. One case series in Belgium reported on 5 adult patients, 43–62 years of age, with Down syndrome, 4 of whom had a severe clinical course; the other was asymptomatic (*28*). In a dual-center study comprising 7,246 COVID-19 patients, including 12 with Down syndrome, levels of inflammation markers, such as C-reactive protein and IL-6, were not much different between 12 patients with Down syndrome and 60 patients without, but the Down syndrome patients had more severe disease (*29*). Nevertheless, the sample size of that study is too small to refute the general belief that IL-6 is a prognostic biomarker for COVID-19. In a study conducted in Sweden, COVID-19 patients with Down syndrome (n = 85) had 1.8 times higher odds of COVID-19 diagnosis and 4.3 times higher odds of ICU admission (*30*). Based on a time-to-event analysis, a study on 8 million adults with SARS-CoV-2 infection reported a 10-fold increase in risk for COVID-19 related hospitalization and 4-fold increase in risk for death among patients with Down syndrome (*31*).

We reduced confounding in our study to enhance the robustness of the results. First, we included several variables, such as intervention and respiratory viral infections, that were not considered in other studies. We also included location variables to account for effects of geographic disparities in access to healthcare. Second, Down syndrome patients have underlying conditions that are also independent risk factors for COVID-19–related death. These conditions are strong confounders between Down syndrome and death, and between Down syndrome and time to recovery. We used a multivariable regression model to reduce confounding, plus we used propensity score matching to confirm the results. Finally, we included cases and controls from the same target population, which increases the level of evidence.

Limitations of this study include missing data, which usually arises in nationwide registries. Using a nationwide database implies large population coverage, but inaccurate data are inevitable. Nevertheless, we made every effort to verify data. Furthermore, we created an additional category for missing data in ethnicity, enabling nonrandom missingness. For missing data in the date of clinical endpoint, we observed many variables that had a statistically significant difference between groups with missing and available data included in the logistic and Cox regression models (Appendix Table 3). This partly accounts for random missingness by conditioning these variables in the regression models. Nonetheless, nonmissing randomness in the date of clinical endpoint remains a limitation. In addition to missing data, we only considered hospitalized cases, limiting the generalizability of our findings. Because predetermined forms were used to standardize the nationwide reporting, no information on clinical management for Down syndrome was available. However, those data are partly reflected in geographic variables that imply access to healthcare. Furthermore, no guidance or details were available on diagnosis of most underlying conditions, except for respiratory viral infections, which were confirmed by PCR; however, data on underlying conditions were registered and verified by certified medical practitioners. Finally, the sample size for the Down syndrome group was small because Down syndrome is a rare disorder, and we only considered a subset of this population, children and adolescents.

In conclusion, our data showed that Down syndrome in children and adolescents is associated with increased risk for severe COVID-19 illness and death among hospitalized patients, even after adjusting for sociodemographic factors and clinical factors common in Down syndrome, such as cardiovascular diseases. Social stratification and the lack of resources at the national level might intensify the risk for severe COVID-19 outcomes among pediatric patients with Down syndrome. Guidelines for managing COVID-19 among Down syndrome patients could improve outcomes for this population.

Appendix Additional information on risk for severe illness and death among pediatric patients with Down syndrome hospitalized for COVID-19, Brazil.

## References

[1] Laignier MR, Lopes-Júnior LC, Santana RE, Leite FMC, Brancato CL. Down syndrome in Brazil: occurrence and associated factors. Int J Environ Res Public Health. 2021;18:11954. 10.3390/ijerph182211954 34831710 PMC8620277

[2] de Campos Gomes F, de Melo-Neto JS, Goloni-Bertollo EM, Pavarino ÉC. Trends and predictions for survival and mortality in individuals with Down syndrome in Brazil: A 21-year analysis. J Intellect Disabil Res. 2020;64:551–60. 10.1111/jir.12735 32378275

[3] Szwarcwald CL, Souza Júnior PR, Marques AP, Almeida WD, Montilla DE. Inequalities in healthy life expectancy by Brazilian geographic regions: findings from the National Health Survey, 2013. Int J Equity Health. 2016;15:141. 10.1186/s12939-016-0432-7 27852270 PMC5112675

[4] Moriyama CH, Mustacchi Z, Pires S, Massetti T, da Silva T, Herrero D, et al. Functional skills and caregiver assistance of Brazilian children and adolescents with Down syndrome. NeuroRehabilitation. 2019;45:1–9. 10.3233/NRE-192763 31450519

[5] Carfì A, Romano A, Zaccaria G, Villani ER, Manes Gravina E, Vetrano DL, et al. The burden of chronic disease, multimorbidity, and polypharmacy in adults with Down syndrome. Am J Med Genet A. 2020;182:1735–43. 10.1002/ajmg.a.61636 32449279

[6] Colvin KL, Yeager ME. What people with Down Syndrome can teach us about cardiopulmonary disease. Eur Respir Rev. 2017;26:160098. 10.1183/16000617.0098-2016 28223397 PMC9489111

[7] Blake JM, Estrada Gomez D, Skotko BG, Torres A, Santoro SL. Pneumonia and respiratory infection in Down syndrome: A 10-year cohort analysis of inpatient and outpatient encounters across the lifespan. Am J Med Genet A. 2021;185:2878–87. 10.1002/ajmg.a.62355 34056836

[8] Kapoor S, Bhayana S, Singh A, Kishore J. Co-morbidities leading to mortality or hospitalization in children with Down syndrome and its effect on the quality of life of their parents. Indian J Pediatr. 2014;81:1302–6. 10.1007/s12098-014-1389-4 24820231

[9] Verstegen RH, van Hout RW, de Vries E. Epidemiology of respiratory symptoms in children with Down syndrome: a nationwide prospective web-based parent-reported study. BMC Pediatr. 2014;14:103. 10.1186/1471-2431-14-103 24735352 PMC4017958

[10] Figliozzi S, Masci PG, Ahmadi N, Tondi L, Koutli E, Aimo A, et al. Predictors of adverse prognosis in COVID-19: A systematic review and meta-analysis. Eur J Clin Invest. 2020;50:e13362. 10.1111/eci.13362 32726868

[11] Emes D, Hüls A, Baumer N, Dierssen M, Puri S, Russell L, et al.; On Behalf Of The Trisomy Research Society Covid-Initiative Study Group. COVID-19 in Children with Down Syndrome: Data from the Trisomy 21 Research Society Survey. J Clin Med. 2021;10:5125. 10.3390/jcm10215125 34768645 PMC8584980

[12] Mesas AE, Cavero-Redondo I, Álvarez-Bueno C, Sarriá Cabrera MA, Maffei de Andrade S, Sequí-Dominguez I, et al. Predictors of in-hospital COVID-19 mortality: A comprehensive systematic review and meta-analysis exploring differences by age, sex and health conditions. PLoS One. 2020;15:e0241742. 10.1371/journal.pone.0241742 33141836 PMC7608886

[13] Dadashi M, Khaleghnejad S, Abedi Elkhichi P, Goudarzi M, Goudarzi H, Taghavi A, et al. COVID-19 and influenza co-infection: a systematic review and meta-analysis. Front Med (Lausanne). 2021;8:681469. 10.3389/fmed.2021.681469 34249971 PMC8267808

[14] Oliveira EA, Colosimo EA, Simões E Silva AC, Mak RH, Martelli DB, Silva LR, et al. Clinical characteristics and risk factors for death among hospitalised children and adolescents with COVID-19 in Brazil: an analysis of a nationwide database. Lancet Child Adolesc Health. 2021;5:559–68. 10.1016/S2352-4642(21)00134-6 34119027 PMC8192298

[15] VanderWeele TJ, Ding P. Sensitivity analysis in observational research: introducing the E-value. Ann Intern Med. 2017;167:268–74. 10.7326/M16-2607 28693043

[16] Dessie ZG, Zewotir T. Mortality-related risk factors of COVID-19: a systematic review and meta-analysis of 42 studies and 423,117 patients. BMC Infect Dis. 2021;21:855. 10.1186/s12879-021-06536-3 34418980 PMC8380115

[17] Tolossa T, Wakuma B, Seyoum Gebre D, Merdassa Atomssa E, Getachew M, Fetensa G, et al. Time to recovery from COVID-19 and its predictors among patients admitted to treatment center of Wollega University Referral Hospital (WURH), Western Ethiopia: Survival analysis of retrospective cohort study. PLoS One. 2021;16:e0252389. 10.1371/journal.pone.0252389 34111146 PMC8191892

[18] Ministério da Saúde (BR). Ordinance no. 793. Establishes the care network for persons with disabilities within the scope of the Unified Health System [in Portuguese]. Brasília: The Ministry; 2012 [cited 2021 Nov 26]. https://bvsms.saude.gov.br/bvs/saudelegis/gm/2012/prt0793_24_04_2012.html

[19] Bermudez BE, Medeiros SL, Bermudez MB, Novadzki IM, Magdalena NI. Down syndrome: Prevalence and distribution of congenital heart disease in Brazil. Sao Paulo Med J. 2015;133:521–4. 10.1590/1516-3180.2015.00710108 26648279 PMC10496550

[20] Huiracocha L, Almeida C, Huiracocha K, Arteaga J, Arteaga A, Blume S. Parenting children with Down syndrome: Societal influences. J Child Health Care. 2017;21:488–97. 10.1177/1367493517727131 29110530 PMC5697561

[21] Allyse M, Minear MA, Berson E, Sridhar S, Rote M, Hung A, et al. Non-invasive prenatal testing: a review of international implementation and challenges. Int J Womens Health. 2015;7:113–26. 10.2147/IJWH.S67124 25653560 PMC4303457

[22] Economic Commission for Latin America and the Caribbean, Pan American Health Organization. The prolongation of the health crisis and its impact on health, the economy and social development. 2021 Oct 14 [cited 2022 Jan 10]. https://iris.paho.org/bitstream/handle/10665.2/54991/eclacpahoreport2021_eng.pdf

[23] Chandrasekharan S, Minear MA, Hung A, Allyse M. Noninvasive prenatal testing goes global. Sci Transl Med. 2014;6:231fs15. 10.1126/scitranslmed.3008704 24718856 PMC4112725

[24] Ferreira CR, de Herreros MB. Medical genetics in paraguay. Mol Genet Genomic Med. 2014;2:458–66. 10.1002/mgg3.119 25614867 PMC4303215

[25] Huggard D, Kelly L, Ryan E, McGrane F, Lagan N, Roche E, et al. Increased systemic inflammation in children with Down syndrome. Cytokine. 2020;127:154938. 10.1016/j.cyto.2019.154938 31785499

[26] Broers CJ, Gemke RJ, Weijerman ME, van der Sluijs KF, van Furth AM. Increased pro-inflammatory cytokine production in Down Syndrome children upon stimulation with live influenza A virus. J Clin Immunol. 2012;32:323–9. 10.1007/s10875-011-9625-4 22170315

[27] Hou Y, Zhao J, Martin W, Kallianpur A, Chung MK, Jehi L, et al. New insights into genetic susceptibility of COVID-19: an ACE2 and TMPRSS2 polymorphism analysis. BMC Med. 2020;18:216. 10.1186/s12916-020-01673-z 32664879 PMC7360473

[28] De Cauwer H, Spaepen A. Are patients with Down syndrome vulnerable to life-threatening COVID-19? Acta Neurol Belg. 2021;121:685–7. 10.1007/s13760-020-01373-8 32444942 PMC7243430

[29] Malle L, Gao C, Hur C, Truong HQ, Bouvier NM, Percha B, et al. Individuals with Down syndrome hospitalized with COVID-19 have more severe disease. Genet Med. 2021;23:576–80. 10.1038/s41436-020-01004-w 33060835 PMC7936948

[30] Bergman J, Ballin M, Nordström A, Nordström P. Risk factors for COVID-19 diagnosis, hospitalization, and subsequent all-cause mortality in Sweden: a nationwide study. Eur J Epidemiol. 2021;36:287–98. 10.1007/s10654-021-00732-w 33704634 PMC7946619

[31] Clift AK, Coupland CAC, Keogh RH, Hemingway H, Hippisley-Cox J. COVID-19 mortality risk in Down syndrome: results from a cohort study of 8 million adults. Ann Intern Med. 2021;174:572–6. 10.7326/M20-4986 33085509 PMC7592804

