# Supplementary material for: Risk for Severe Illness and Death among Pediatric Patients with Down Syndrome Hospitalized for COVID-19, Brazil
Source: Emerg Infect Dis. 2023 Jan;29(1):26–35. doi: 10.3201/eid2901.220530 (PMC9796221; doi:10.3201/eid2901.220530)
Supplement: Appendix — Additional information on risk for severe illness and death among pediatric patients with Down syndrome hospitalized for COVID-19, Brazil. [file 22-0530-Techapp-s1.pdf]

Article DOI: https://doi.org/10.3201/eid2901.220530

Risk for Severe Illness and Death among Pediatric Patients with Down syndrome Hospitalized for COVID-19, Brazil

Appendix

Appendix Table 1. E-values for multivariable logistic regression in a study of risk for severe illness and death among pediatric patients with Down syndrome hospitalized for COVID-19, Brazil

Characteristics	E-value
Down syndrome	3.5
Location: Northeast	5.8
Cardiovascular disease	5.5
Neurologic disease	5.9
Immunocompromised	5.0
Location: North	4.5
Liver disease	7.8
Renal disease	3.2
Ethnicity: Indigenous	7.9
Pulmonary disease	3.5
Hematologic disease	2.5
Obesity	4.0
Asthma	3.5
Vaccination against influenza	3.3
Age	1.2
Male sex	1.6

Appendix Table 2. E-values for multivariable Cox regression in a study of risk for severe illness and death among pediatric patients with Down syndrome hospitalized for COVID-19, Brazil

Characteristics	E-value
Down syndrome	4.3
Neurologic disease	4.3
Location: Northeast	2.3
Immunocompromised	2.9
Cardiovascular disease	3.8
Asthma	2.2
Location: North	2.4
Renal disease	8.2
Pulmonary disease	1.6
Hematologic disease	4.1
Vaccination against influenza	1.7
Liver disease	11.8
Obesity	2.1
Use of antiviral	1.0
Diabetes	3.2
Ethnicity: Indigenous	2.6
Location: Center West	1.3
Ethnicity: Asian	1.4

Appendix Table 3. Difference in characteristics of cohort (between those with and without the date of clinical endpoint) in a study of risk for severe illness and death among pediatric patients with Down syndrome hospitalized for COVID-19, Brazil*

	Date of clinical endpoint		
Characteristics	Missing, n = 1,489	Available, n = 13,195	p value
Median age, y (IQR)	4.9 (0.9–13.3)	3.5 (0.7–11.3)	<0.001
Sex, no. (%)		N = 13,188	
M	761 (51.1)	7, 136 (54.1)	0.030
F	727 (48.9)	6,052 (45.9)	
Died, no. (%)	133 (8.99)	1,104 (8.4)	0.460
Median time to recover, d (IQR)	NA	5.0 (3.0–10.0), n = 12,091	NA
Region, no. (%)			
North	142 (9.5)	782 (5.9)	<0.001
Northeast	501 (33.6)	2,891 (21.9)	<0.001
Southeast	521 (35.0)	6,487 (49.2)	<0.001
Center West	252 (16.9)	1,101 (8.3)	<0.001
South	73 (4.9)	1,934 (14.7)	<0.001
Ethnicity, no. (%)	N = 1,194	N = 10,207	
Caucasian	413 (34.6)	4,588 (44.9)	<0.001
Asian	18 (1.5)	70 (0.7)	0.005
Hispanic	750 (62.8)	5,482 (53.7)	<0.001
Indigenous	13 (1.1)	67 (0.7)	0.098
Missing	295 (19.8), n = 1,489	2,988 (22.6), n = 13,195	0.013
Signs and symptoms, no. (%)			
Asymptomatic	8 (0.5)	53 (0.4)	0.397
Abdominal pain	55 (3.7)	841 (6.4)	<0.001
Anosmia	47 (3.2)	259 (2.0)	0.004
Ageusia	41 (2.8)	257 (1.9)	0.041
Coryza	158 (10.6)	1,331 (10.1)	0.526
Cough	900 (60.4)	8,143 (61.7)	0.339
Diarrhea	197 (13.2)	1,830 (13.9)	0.526
Dyspnea	609 (40.9)	6,591 (50.0)	<0.001
Fatigue	100 (6.7)	1,173 (8.9)	0.004
Fever	973 (65.3)	8,866 (67.2)	0.154
Headache	141 (9.5)	665 (5.0)	<0.001
Myalgia	62 (4.2)	326 (2.5)	<0.001
Oxygen saturation <95%	365 (24.5)	5,174 (39.2)	<0.001
Respiratory discomfort	535 (35.9)	6,218 (47.1)	<0.001
Sore throat	304 (20.4)	1,668 (12.6)	<0.001
Vomiting	218 (14.6)	2,270 (17.2)	0.012
Other symptoms	527 (35.4)	4,739 (35.9)	0.711
Underlying conditions, no. (%)			
Cardiovascular disease	58 (3.9)	531 (4.0)	0.889
Hematologic disease	17 (1.1)	261 (2.0)	0.027
Down syndrome	13 (0.9)	223 (1.7)	0.016
Liver disease	5 (0.3)	65 (0.5)	0.551
Asthma	70 (4.7)	993 (7.5)	<0.001
Diabetes	23 (1.5)	265 (2.0)	0.277
Neurologic disease	40 (2.7)	785 (5.9)	<0.001
Pulmonary disease	21 (1.4)	291 (2.2)	0.046
Immunocompromised	39 (2.6)	490 (3.7)	0.033
Renal disease	18 (1.2)	177 (1.3)	0.811
Obesity	16 (1.1)	314 (2.4)	0.001
Intervention, no. (%)			
Antiviral against influenza	108 (7.3)	1,481 (11.2)	<0.001
ICU admission	156 (10.5)	3,860 (29.3)	<0.001
Ventilation	330 (22.2)	6,255 (47.4)	<0.001
Vaccination against influenza	88 (5.9)	911 (6.9)	0.158
Vaccination against SARS-CoV-2	8 (0.5)	76 (0.6)	>0.999

*ICU, intensive care unit; NA, not applicable.
